# Hepatic glucose utilization in hepatic steatosis and obesity

**DOI:** 10.1042/BSR20160381

**Published:** 2016-11-03

**Authors:** Georgia Keramida, James Hunter, Adrien Michael Peters

**Affiliations:** * Clinical Imaging Sciences Centre, Brighton and Sussex Medical School, Brighton BN1 9PX, U.K.; † Division of Clinical and Laboratory Investigation, Brighton and Sussex Medical School, Brighton BN1 9PX, U.K.

**Keywords:** F-18-fluorodeoxyglucose (FDG), hepatic glucose utilization, hepatic steatosis, liver, PET/CT

## Abstract

Hepatic steatosis is associated with obesity and insulin resistance. Whether hepatic glucose utilization rate (glucose phosphorylation rate; MRglu) is increased in steatosis and/or obesity is uncertain. Our aim was to determine the separate relationships of steatosis and obesity with MRglu. Sixty patients referred for routine PET/CT had dynamic PET imaging over the abdomen for 30 min post-injection of F-18-fluorodeoxyglucose (FDG), followed by Patlak–Rutland graphical analysis of the liver using abdominal aorta for arterial input signal. The plot gradient was divided by the intercept to give hepatic FDG clearance normalized to hepatic FDG distribution volume (ml/min per 100 ml) and multiplied by blood glucose to give hepatic MRglu (μmol/min per 100 ml). Hepatic steatosis was defined as CT density of ≤40 HU measured from the 60 min whole body routine PET/CT and obesity as body mass index of ≥30 kg/m^2^. Hepatic MRglu was higher in patients with steatosis (3.3±1.3 μmol/min per 100 ml) than those without (1.7±1.2 μmol/min per 100 ml; *P*<0.001) but there was no significant difference between obese (2.5±1.6 μmol/min per 100 ml) and non-obese patients (2.1±1.3 μmol/min per 100 ml). MRglu was increased in obese patients only if they had steatosis. Non-obese patients with steatosis still had increased MRglu. There was no association between MRglu and chemotherapy history. We conclude that MRglu is increased in hepatic steatosis probably through insulin resistance, hyperinsulinaemia and up-regulation of hepatic hexokinase, irrespective of obesity.

## INTRODUCTION

**Figure 1:**
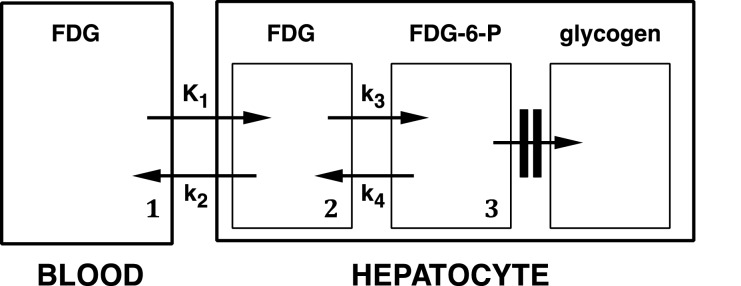
Model of FDG kinetics with reference to the liver *K*_1_ is hepatic blood flow, *k*_2_ is a diffusion constant, *k*_3_ is hexokinase and *k*_4_ is glucose-6-phosphatase. FDG is assumed to mix throughout its intrahepatic distribution volume (compartments 1 and 2) via *K*_1_ and *k*_2_ by 2 min post-injection. De-phosphorylation (via *k*_4_) in compartment 3 is assumed to be slow enough to ignore. Patlak–Rutland analysis therefore measures *k*_3_.

Recent interest in the hepatic accumulation of the glucose radiotracer, F-18-fluorodeoxyglucose (FDG), has focused on three issues: firstly, the validity of using the steatotic liver as a reference region for tumour FDG uptake in PET/CT [1–5]; secondly, the possible use of FDG PET/CT to diagnose hepatic inflammation, especially non-alcoholic steatohepatitis (NASH) [5,6] and thirdly, the determinants of hepatic glucose utilization (MRglu; i.e. hepatic phosphorylation rate of glucose to glucose-6-phosphate via hexokinase; transport constant *k*_3_; Figure 1), especially in hepatic steatosis, insulin resistance and metabolic syndrome [7–9].

Choi et al. [7] were the first to use FDG PET to study hepatic glucose kinetics. They showed a dramatic increase in MRglu (metabolic rate for glucose) following acute administration of glucose in healthy subjects, an increase that was presumably mediated by insulin acting on hepatic hexokinase (*k*_3_). Iozzo et al. [8], employing the euglycaemic hyperinsulinaemic clamp, confirmed that insulin increases hepatic phosphorylation of FDG to FDG-6-phosphate (FDG-6-P). Interestingly, they showed that the increase in *k*_3_ in response to hyperinsulinaemia was at least as marked in patients with low insulin sensitivity as in healthy sedentary subjects (normal insulin sensitivity) and athletes (high insulin sensitivity), suggesting that *k*_3_ remains sensitive to insulin in patients with insulin resistance. In contrast, the de-phosphorylation rate of FDG-6-P by glucose-6-phosphatase (*k*_4_) was very slow and insensitive to hyperinsulinaemia in patients with reduced insulin sensitivity.

Insulin resistance is strongly associated with obesity, type 2 diabetes mellitus and hepatic steatosis [10–16]. Patients with insulin resistance have raised blood insulin levels [17]. It is therefore surprising that Borra et al. [9] subsequently found hepatic glucose uptake to correlate *inversely* with hepatic steatosis in patients with type 2 diabetes and to be higher in normal subjects compared with type 2 diabetics.

Although an association between obesity, hepatic steatosis and insulin resistance is well established, how the three conditions are linked remains uncertain. There is strong evidence to suggest that insulin resistances and hepatic steatosis are driven by obesity-induced adipokines [18]. However, not all patients with hepatic steatosis are obese and not all obese patients have hepatic steatosis [19,20]. No previous study has separated the relationships of obesity and hepatic steatosis with MRglu. The purpose of the present study therefore was to examine the relationship between hepatic steatosis and MRglu and determine the separate relationships of obesity and hepatic steatosis with MRglu.

## MATERIALS AND METHODS

### Patients

Sixty patients (47 men, age range 28–84, and 13 women, age range 40–67) having routine, clinical PET/CT, mostly for the management of cancer, were prospectively recruited for the study. These patients formed the population for a study on a separate issue concerning signal-to-noise ratio in PET published elsewhere [21]. They were classified as obese if body mass index was ≥30 kg/m^2^ and as having hepatic steatosis if CT density was ≤40 HU [22], as determined from the CT component of their clinical PET/CT study. Twelve had metabolically active lymphoma, 8 had inactive lymphoma, 26 had FDG-avid non-haematological malignancy and 14 more had normal PET/CT. Twelve had received chemotherapy within 6 months of their scan, 19 had received chemotherapy >6 months previously (range 8 months to 10 years) and 29 patients had received no previous chemotherapy (chemotherapy-naïve). Five patients had type 2 diabetes mellitus. There were none with type 1 diabetes mellitus. Patients with known or suspected high ethanol intake were not included. There were a total of 16 patients with no FDG avid malignancy, no hepatic steatosis and no recent chemotherapy, considered to be ‘almost normal’. Ethical approval was given by a National Research Ethics Committee and all patients gave written informed consent.

### Imaging

Patients fasted for 6 h. Blood glucose was measured immediately before FDG injection using a glucometer (ACCU-CHEK Performa; Inform ll strips; Roche). Prior to routine whole body imaging, dynamic PET imaging was performed following i.v. injection of 400 MBq (±10%) FDG, acquiring 30×1 min frames, using a Siemens Biograph 64-slice 16 Truepoint PET/CT scanner (Erlangen, Germany). Following the dynamic study, the patient had routine PET/CT at 60 min post-injection; 3D emission data were acquired at 3 min per bed position (PET reconstruction: four iterations; subset 8; Gaussian pre-filter; FWHM 5 mm; matrix size 168×168; zoom 1).

### Image analysis

Hepatic clearance was measured from the dynamic data using Patlak–Rutland analysis. Using *Hermes* software (HERMES), liver activity was summed from regions of interest (ROI) of 3 cm diameter each on approximately 20 transaxial images, avoiding any suspected focal pathology in each transaxial image. Blood pool activity was obtained from ROIs of 1.6 cm diameter carefully placed within the wall of the abdominal aorta in each of approximately 20 transaxial images. Other workers have validated the use of the abdominal aorta for Patlak–Rutland analysis [23,24], including the liver [23].

### Patlak–Rutland graphical analysis

**Figure 2:**
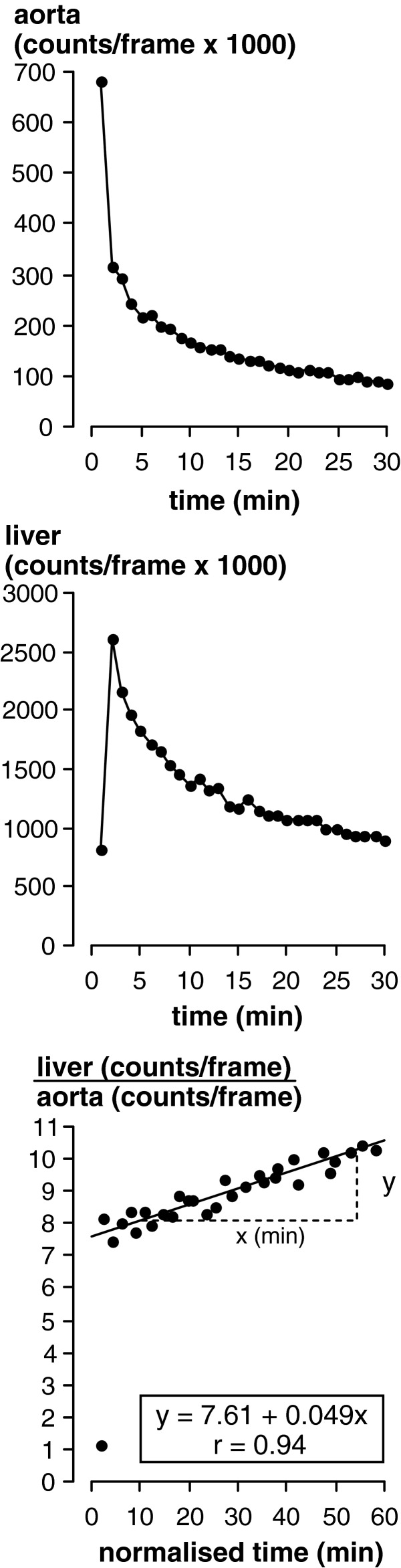
Examples of time compared with counts/frame curves for the aortic blood pool (top), liver (middle) and Patlak–Rutland plot based on these curves (bottom) Note that the gradient of the plot (Δ*y*/Δ*x*) is proportional to FDG clearance with the same proportionality constant as that relating the intercept to the distribution volume.

After correction for physical decay of F-18, the ratio of hepatic-to-aortic counts/frame was plotted against the ratio of integral of aortic counts-to-aortic counts/frame (Figure 2). The latter ratio has units of time (‘normalized time’). The gradient of the plot is proportional to hepatic FDG clearance (Ki) and the intercept is proportional to the distribution volume of un-phosphorylated FDG throughout the liver (*V*(0)) with the same proportionality constant (see Appendix A). Ki was divided by *V*(0) to give hepatic FDG clearance per unit FDG hepatic distribution volume. In healthy liver, *V*(0) is almost unity [7,8,25], indicating that FDG rapidly penetrates not only the hepatic interstitial space but also hepatocytes. Ki/*V*(0) is therefore effectively FDG clearance per unit volume of lean liver and, according to standard equations linking *K*_1_, *k*_2_, *k*_3_, *k*_4_, Ki and *V*(0), is equal to *k*_3_ [7,8,25]. FDG does not penetrate hepatic fat, the presence of which consequently physically dilutes the FDG signal [26]. Hepatic fat may account for up to 30% of the liver (equivalent to CT density of ∼5 HU [26]) in which case hepatic FDG clearance measured as Ki would underestimate clearance into lean liver by 30%. Moreover, the distribution of hepatic fat is heterogeneous [27]. Expressing clearance as Ki/*V*(0) is therefore desirable for determining the relationship between hepatic MRglu and hepatic steatosis. Ki/*V*(0) was multiplied by the blood glucose concentration (μmol/ml) to give MRglu in units of μmol/min per 100 ml, where 100 ml represents 100 ml fat-free liver. We assumed a lumped constant of unity [28].

Patlak–Rutland analysis is valid when transport of tracer is unidirectional along a single transport pathway. Mixing of FDG between intrahepatic blood and hepatocytes (compartments 1 and 2, Figure 1) is rapid because *K*_1_ and *k*_2_ are high [7,8,25]. Conversely, the de-phosphorylation rate of FDG-6-P via glucose-6-phosphatase appears to be very slow [8,25]. So the only effective transport constant is *k*_3_, which reflects the clearance of FDG by conversion to FDG-6-P.

Mixing of FDG between compartments 1 and 2 was assumed to have been completed within 2 min of FDG injection so the first 2 frame values were not included in the Patlak–Rutland plot. Inspection of the plots revealed that they were essentially linear from 3 to 30 min (Figure 2), consistent with this assumption. This mixing time may seem short but is consistent with previously reported values of *K*_1_ and *k*_2_, which respectively range from 0.01 to 0.015 and 0.013 to 0.016 s^−1^ [7,8,25], and which therefore give an equilibration rate constant of 0.023–0.031 s^−1^. This gives a time to 95% equilibration of FDG between compartments 1 and 2 of 97–130 s. Munk et al. [25] also obtained Patlak–Rutland plots that were linear within a very few minutes of injection.

### Statistical analysis

Normal distributions of data were confirmed using the Shapiro–Wilk test, so parametric statistics were used. Values were expressed as mean ± S.D. Correlations were quantified using Pearson's analysis. Significance of differences between patient groups was tested using Student's unpaired *t* test.

## RESULTS

Patient demographics are briefly summarized in Table 1.

**Table 1:** Patient demographics (±S.D. where indicated)

	CT density (HU)		BMI (kg/m^2^)	
	≤40	>40	<30	≥30
Male/female	16/3	32/9	32/10	16/2
Age (years)	60±13	60±12	62±12	57±13
Blood glucose (mmol/l)	6.4±1.5	5.7±0.6	6.3±1.5	5.7±0.7
Obese/non-obese	11/8	7/34	–	–
Steatosis/no steatosis	–	–	8/34	11/7
PET FDG-avid/non-avid	13/6	21/20	23/20	11/6

### Effect of chemotherapy

Mean CT density in 29 chemotherapy-naïve patients was 46±9 HU compared with 43±9 HU in 12 with a history of recent chemotherapy (*P*=0.33). Patients with distant chemotherapy had a mean CT density of 47±11 HU (*P*=0.8 compared with chemotherapy-naïve patients). Corresponding values of BMI were 28±5, 28±8 and 26±4 kg/m^2^ (*P* > 0.1). There was no significant difference in MRglu between patients with recent chemotherapy (2.6±1.3 μmol/min per 100 ml) and chemotherapy-naïve patients (2.3±1.4 μmol/min per 100 ml; *P*=0.52).

### Prevalence of hepatic steatosis in obese and non-obese patients

**Figure 3:**
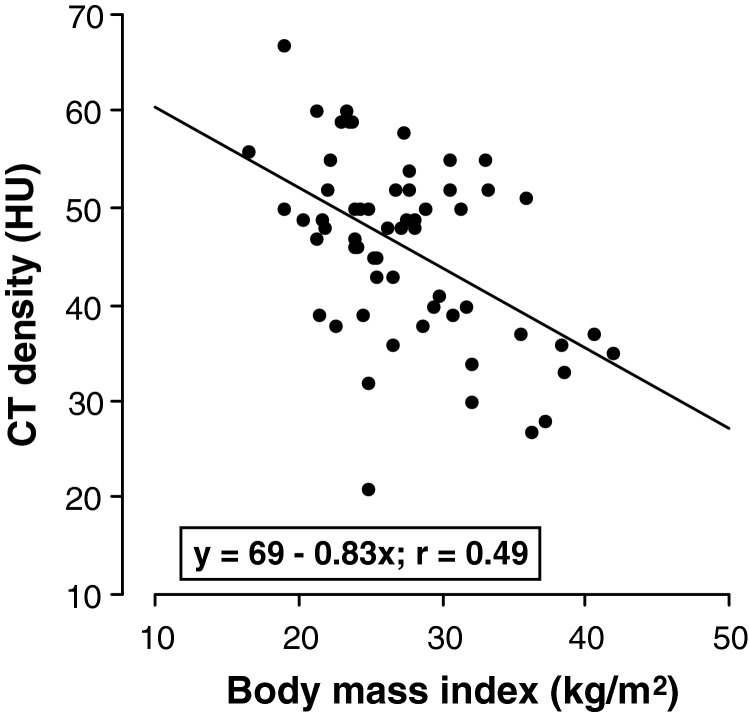
Relationship between CT density and BMI in all patients

CT density correlated inversely with BMI (CT density=69–0.83*BMI; *r*=0.49; *P*=0.0001; Figure 3). Of the 60 patients, 19 (32%) had steatosis and 18 (30%) were obese. Thirty-four had neither steatosis nor obesity, whereas 11 had both. Of the 19 with steatosis, 8 (42%) were not obese, whereas of the 18 who were obese, 11 (61%) had steatosis (Table 1).

### Blood glucose levels

Blood glucose levels were slightly but significantly higher in patients with steatosis (6.4±1.5 mmol/l) compared with those without (5.7±0.6 mmol/l; *P*=0.012) and in obese subjects (6.3±1.5 mmol/l) compared with non-obese (5.7±0.7 mmol/l; *P*=0.035; Table 1).

### Hepatic FDG clearance and MRglu

Liver and aortic blood pool time-activity curves followed similar time courses (Figure 2). Relative to the intercept, Patlak–Rutland analysis of dynamic hepatic and aortic blood pool activity therefore generated fairly shallow positive gradients that appeared to be essentially linear from 3 to 30 min.

**Figure 4:**
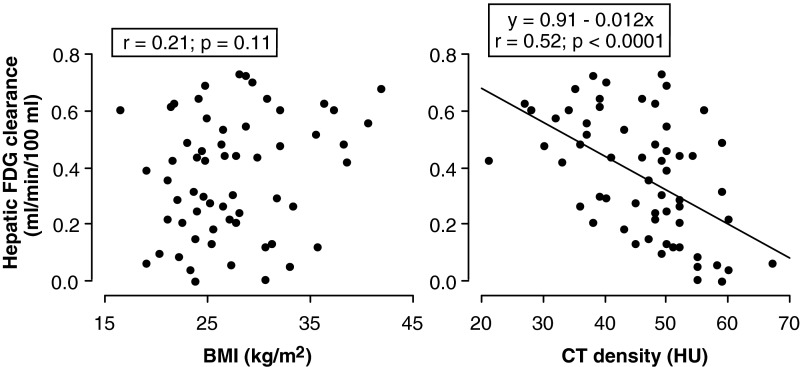
Relations of hepatic FDG clearance with BMI (left panel) and CT density (right panel)

**Figure 5:**
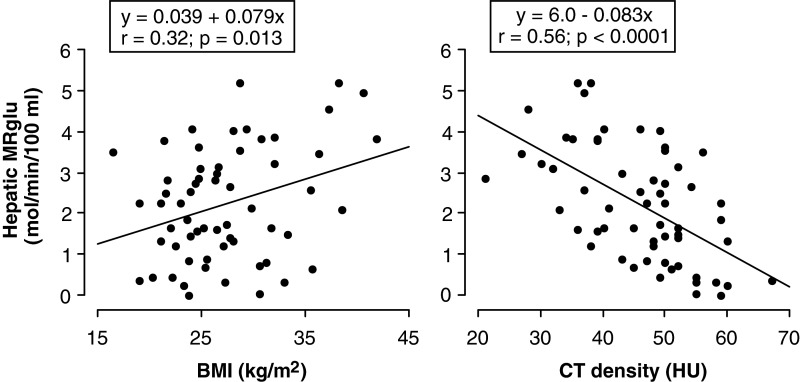
Relations of hepatic glucose utilization rate (MRglu) with BMI (left panel) and CT density (right panel)

Mean MRglu in the almost normal 16 patients was 1.6±1.2 μmol/min per 100 ml. In all 60 patients, there were strong negative correlations between CT density and FDG clearance (*r*=−0.52; *P*<0.0001) and between CT density and MRglu (*r*=−0.56; *P*<0.0001; Figures 4 and 5). In contrast, BMI correlated weakly but significantly with MRglu (*r*=0.32; *P*=0.013) and showed an insignificant correlation with clearance (*r*=0.21; *P*=0.11).

**Figure 6:**
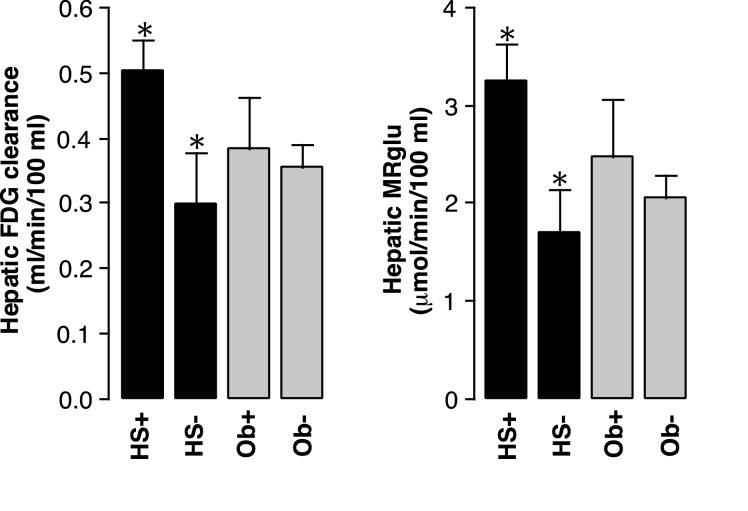
Hepatic FDG clearance and MRglu are increased in patients with hepatic steatosis (HS+) compared with those without (HS−) In contrast, there are no significant differences between obese (Ob+) and non-obese (Ob−) patients (*P<0.001; bars=S.E.M.).

**Figure 7:**
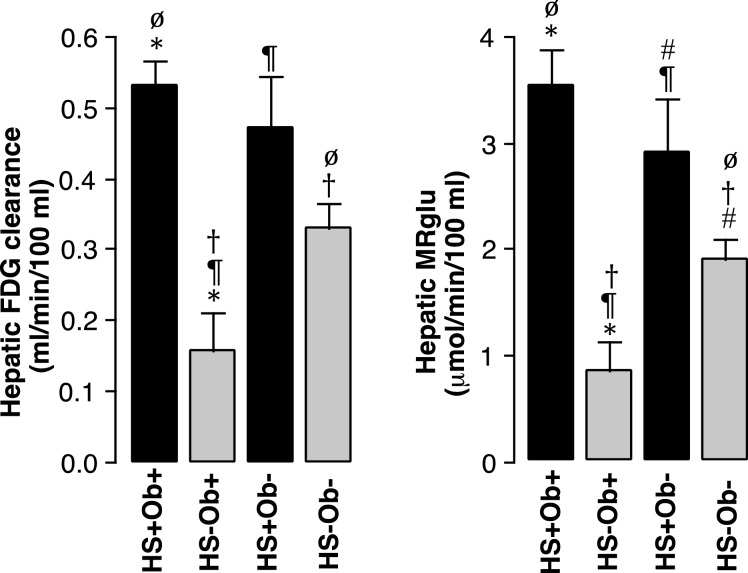
Hepatic FDG clearance and MRglu are increased in patients with steatosis (HS+) whether or not they are obese (Ob+) In contrast, obese patients only have increased clearance and MRglu when they also have steatosis (*P<0.001; ^ø^*P*<0.01; ^¶^*P*<0.01; ^#^*P*<0.05; ^†^*P*<0.05; symbols identify paired columns for *t* test; bars=S.E.M.).

Consistent with the above correlations, MRglu was higher in patients with steatosis (3.3±1.3; *n*=19) than in those without (1.7±1.2; *n*=41; *P*<0.001) but the difference between obese (2.5±1.6; *n*=18) and non-obese patients (2.1±1.3; *n*=42) was not significant (*P*=0.2) (Figure 6). There was no significant difference in MRglu between 8 non-obese (2.9±1.4) and 11 obese (3.5±1.1) patients with steatosis (*P*=0.27) (Figure 7). However, MRglu in these 11 obese patients with steatosis was higher than in 7 obese patients without steatosis (0.8±0.7; *P*<0.001). Similar results were obtained with respect to FDG clearance instead of MRglu (Figures 6 and 7). Thus, clearances were 0.51±0.16 ml/min per 100 ml in steatosis compared with 0.30±0.21 ml/min per 100 ml in patients without steatosis (*P*<0.001) and 0.39±0.23 ml/min per 100 ml in obese patients compared with 0.36±0.21 ml/min per 100 ml in non-obese patients (*P*=0.62).

Of five patients with type 2 diabetes mellitus, four, all with steatosis (CT densities: 36, 37, 37 and 38 HU), had high values of FDG clearance (0.57±0.11 ml/min per 100 ml) and MRglu (4.5±1.3 μmol/min per 100 ml). In contrast, one patient without steatosis (CT density 50 HU) had low values (0.13 ml/min per 100 ml and 0.8 μmol/min per 100 ml respectively).

## DISCUSSION

Hepatocytes, but not other cells, contain glucose-6-phosphatase, which de-phosphorylatesglucose-6-phosphate and FDG-6-P via pathway *k*_4_ (Figure 1). In the fasting state, hepatic glucose production exceeds hepatic glucose uptake as a result of hepatic glycogenolysis and the production of glucose-6-phosphate, thereby maintaining the blood glucose level. FDG, however, is not incorporated into glycogen so the hepatic FDG-6-P concentration depends on the balance of *k*_3_ and *k*_4_. Based on modelling of hepatic FDG kinetics, *k*_4_ has been shown to be low [8] or indeterminate [7] in the fasting state. Dephosphorylation of FDG-6-P is therefore slow and this is compounded by the relatively long time required, via hexokinase, to generate sufficient phosphorylated FDG for *k*_4_ to be clearly detectable. FDG clearance and MRglu are therefore effectively determined by *k*_3_. Previous studies using Patlak–Rutland analysis to measure hepatic glucose utilization rate [8,9,25] recorded linear plots, as we have done, confirming a very low rate of FDG de-phosphorylation. Moreover, these studies found Patlak–Rutland analysis to be robust for measuring MRglu and independent of errors arising from the liver's dual blood supply that complicate measurement of the transport constants using modelling [25,29].

We measured MRglu by multiplication with blood glucose rather than plasma glucose. Ki and *V*(0) are lower when based on plasma sampling than whole blood but Ki/*V*(0) is the same. However, the distribution ratio of FDG between erythrocytes and plasma exceeds 0.8 [30] and plasma glucose is only approximately 10% higher than blood glucose. This means that plasma FDG clearance is only approximately 10% lower than blood clearance and *V*(0) based on plasma sampling is similarly less than when derived from blood sampling. To compare our values of MRglu with literature values, we need therefore to take into account that previous studies expressed Ki as blood clearance and some as plasma clearance. We also need to take into account that previous studies expressed Ki in terms of total liver volume, including fat. Notwithstanding these limitations, our mean value of MRglu in almost healthy patients of 1.6 μmol/min per 100 ml is similar to values in healthy subjects reported by Choi et al. [7] (2.1 μmol/min per 100 ml; *n*=10) and Iozzo et al. [8] (1.3 μmol/min per 100 ml; *n*=16), but less than that of Borra et al. [9] who obtained a mean value in eight healthy subjects of 3.6 μmol/min per100 ml. Choi et al. [7] found *V*(0) to be 0.88 ml/g in healthy subjects, both fasting and after a glucose load, whereas Iozzo et al. recorded a value in healthy subjects of ∼0.8 ml/ml. *V*(0), however, will be lower in hepatic steatosis because fat will increase *V* relative to *V*(0). Interestingly, in the study of Iozzo et al. [8], *V*(0) was lower in subjects with low insulin sensitivity, 0.75 ml/ml, perhaps because they had higher hepatic fat burden. Munk et al. [25] performed Patlak–Rutland analysis in pigs and obtained *V*(0) of 1.05 ml/ml but they appeared to count whole blood rather than plasma.

Dividing Ki by *V*(0) has the advantage that it reflects MRglu in terms of lean hepatic volume and avoids the issues of fat signal dilution [26], fat distribution heterogeneity [27] and blood compared with plasma clearance. Jones et al. [31] and Subramanian et al. [32] also expressed FDG clearance in terms of distribution volume in the lungs in patients with chronic obstructive pulmonary disease, where there are similar problems because of variations in airspace volume in COPD (cf. fat in the liver).

The duration of our dynamic acquisition may be considered limited but longer acquisition periods risk patient movement artefacts, especially in patient volunteers rather than motivated normal volunteers [7–9], and a possible influence of *k*_4_ as intrahepatic FDG-6-P increases, so 30 min seemed reasonable. Others used 40 min [8,9] or 60 min [7].

Physiological hyperinsulinaemia, mediated through an acute glucose load, increases the liver-to-blood FDG concentration ratio in normal subjects [7]. Hepatic steatosis is associated with insulin resistance [10–16]. According to the data of Iozzo et al. [8], *k*_3_, but not *k*_4_, appears unaffected by insulin resistance. Thus, they showed that compared with fasting values, *k*_3_ increased during euglycaemic hyperinsulinaemic clamp in patients with reduced insulin sensitivity as much as in subjects with normal insulin sensitivity. So in insulin resistance, hexokinase is up-regulated as a result of hyperinsulinaemia and increases hepatic glucose clearance. Conversely, glucose-6-phosphatase is insensitive to insulin in insulin resistance. Thus Iozzo et al. [8] found that during hyperinsulinaemia, *k*_4_ was much higher in patients with low insulin sensitivity compared with those with normal or high sensitivity. So in fasting subjects with insulin resistance, *k*_3_ and *k*_4_ are *both* up-regulated. Up-regulation of *k*_3_ is therefore the probable explanation for the increased MRglu of steatosis. An alternative explanation is increased FDG uptake in metabolically active intrahepatic leucocytes in patients with undiagnosed NASH. This is unlikely however because NASH affects only approximately 10% of patients with hepatic steatosis [33], meaning that only two or three patients in our population would have had NASH.

In the only other study we are aware of to measure MRglu in hepatic steatosis by dynamic FDG PET imaging [9], an inverse association between hepatic fat content and glucose utilization was found in patients with type 2 diabetes mellitus, contradicting our findings. However, they measured hepatic FDG concentration in absolute units so total liver volume, against which MRglu was expressed, included fat, which may partly explain this discrepancy. Nevertheless, the range of MRglu they reported is strikingly similar to the range in our five diabetics.

Although obesity is associated with insulin resistance, we found that MRglu was not increased in obese individuals who did not have steatosis. Conversely, non-obese patients with steatosis had increased MRglu. These data show that steatosis, and not obesity *per se,* is associated with increased glucose utilization and probably with insulin resistance.

Non-alcoholic fatty liver disease (NAFLD) is very common and generally thought to be a complication of obesity. It is estimated that the prevalence of NAFLD in Caucasians is approximately 75% in obese subjects [14] and approximately 15% in non-obese subjects [19]. The overall prevalence of obesity is 20–25%, so the prevalence of NAFLD in the general population is approximately 30%. Of our 60 patients, 19 (32%) had steatosis. However, although our patients were not healthy and their numbers small, we found that 42% of these 19 with steatosis were not obese, a rather high value that cannot be attributed to chemotherapy.

In conclusion, hepatic glucose utilization is increased in hepatic steatosis, independently of obesity, probably as a result of insulin resistance, hyperinsulinaemia and up-regulation of hepatic hexokinase.

## APPENDIX A

According to the theory of the Patlak–Rutland plot

1 $$\frac{\mathrm{FDG}\left(t\right)+\mathrm{FDG}6P\left(t\right)}{C\left(t\right)}\;=\mathrm{Ki}\cdot T+\frac{\mathrm{FDG}\left(t\right)}{C\left(t\right)}$$

where FDG and FDG6P are un-phosphorylated and phosphorylated FDG concentrations in the liver, *C* is FDG concentration in blood and *T* is normalized time [∫*C*(*t*)ċd*t*/*C*(*t*)]. Note that FDG(*t*)/*C*(*t*) is *V*(0) and the intercept of the plot.

Re-arranging eqn (1)

2 $$\frac{\mathrm{FDG}6P\left(t\right)+\mathrm{FDG}\left(t\right)}{C\left(t\right)}-\frac{\mathrm{FDG}\left(t\right)}{C\left(t\right)}=\mathrm{Ki}\cdot T$$

Dividing through by FDG(*t*)/*C*(*t*) [i.e. *V*(0)],

3 $$\frac{\left[\mathrm{FDG}6P\left(t\right)+\mathrm{FDG}\left(t\right)\right]/C\left(t\right)-\left[\mathrm{FDG}\left(t\right)\right]/C\left(t\right)}{\mathrm{FDG}\left(t\right)/C\left(t\right)}=\frac{\mathrm{Ki}\cdot T}{V\;\;\left(0\right)}$$

Let *h* and *a* be the proportionality constants relating hepatic and arterial tracer concentrations to hepatic (*H*_FDG_ and *H*_FDG6P_) and abdominal aortic (*A*) counts, respectively, then

4 $$\begin{array}{ccc} & & \frac{\left[h\cdot \left\{H_{\mathrm{FDG}}\left(t\right)+H_{\mathrm{FDG}6P}\left(t\right)\right\}/a\cdot A\left(t\right)\right]-\left[h\cdot H_{\mathrm{FDG}}\left(t\right)/a\cdot A\left(t\right)\right]}{h\cdot H_{\mathrm{FDG}}\left(t\right)/a\cdot A\left(t\right)}\\ & & \;=\frac{Ki\cdot T}{V\left(0\right)} \end{array}$$

*h* and *a* cancel out so

5 $$\frac{\left[\left\{H_{\mathrm{FDG}}\left(t\right)+H_{\mathrm{FDG}6P}\left(t\right)\right\}/A\left(t\right)\right]-\left[H_{\mathrm{FDG}}\left(t\right)/A\left(t\right)\right]}{T\cdot \left[H_{\mathrm{FDG}}\left(t\right)/A\left(t\right)\right]}\;\frac{\mathrm{Ki}}{V\;\;\left(0\right)}$$

([{*H*_FDG_(*t*) + *H*_FDG6P_(*t*)}/*A*(*t*)] − [*H*_FDG_(*t*)/*A*(*t*)])*T* is the gradient of the plot based on raw counts and *H*_FDG_(*t*)/*A*(*t*) is the intercept. Therefore, by dividing the gradient by the intercept, ROI of any size can be used for the Patlak–Rutland analysis and there is no need for attenuation correction of either hepatic or abdominal aortic counts.

## Abbreviations

FDG: F-18-fluorodeoxyglucose
FDG-6-P: FDG-6-phosphate
NAFLD: non-alcoholic fatty liver disease
NASH: non-alcoholic steatohepatitis
ROI: regions of interest
